# Challenges in global reconstructive microsurgery: The sub-Saharan african surgeons’ perspective

**DOI:** 10.1016/j.jpra.2019.01.009

**Published:** 2019-02-04

**Authors:** Chihena H. Banda, Pafitanis Georgios, Mitsunaga Narushima, Ryohei Ishiura, Minami Fujita, Jovic Goran

**Affiliations:** a Department of Plastic and Reconstructive Surgery, Graduate School of Medicine, Mie University, Tsu, Japan; b Department of Surgery, Arthur Davison Children's Hospital, Ndola, Zambia; c Group for Academic Plastic Surgery, The Royal London Hospital, Barts Health NHS Trust, Queen Mary University of London, London, UK; d Department of Surgery, The University Teaching Hospital, Lusaka, Zambia

**Keywords:** Microsurgery, Free tissue transfer, Africa, Challenges, Training, Global surgery

## Abstract

**Background:**

Microsurgery is an essential element of plastic surgery practice. However, it remains unavailable or rudimentary in several developing countries, especially in sub-Saharan Africa. This study presents the local plastic surgeons experience, while focusing on specific challenges encountered and methods to improve the sub-Saharan global microsurgery practice.

**Methodology:**

An online survey was sent to all plastic surgeons registered with the College of Surgeons East Central and Southern Africa and respective national plastic surgical societies in the east central and southern Africa regional community. A total of 57 questionnaires were sent. Surgeons' country of practice, years of experience and rate of performing microsurgical procedures were considered.

**Results:**

The survey response rate was 56% (*n* = 32). Most participants believed microsurgery was essential in the region. The leading challenge was inadequate perioperative care, mainly attributed to shortage of support staff (*n* = 29, 91%). Others were lack of surgical expertise and resources. Interestingly, public unawareness of the benefits of microsurgery was also noted as a critical hindrance. The foremost suggestion on improvement (*n* = 19, 59%) was to enhance training with a multidisciplinary team-building approach. Others included increased advocacy, publications and funding.

**Conclusion:**

The Plastic surgeons' perspective recognizes the needs of Global Reconstructive Microsurgery in sub-Saharan Africa. However, inadequate perioperative care, insufficient expertise, lack of equipment and lack of public awareness were major hindrances. Finally, there is a need to improve microsurgery in the region through advocacy, training and multidisciplinary team building.

## Introduction

Microsurgery is an essential component of modern plastic reconstructive surgery. The technique facilitates free tissue transfer providing optimal functional and aesthetic recovery for a wide range of complex tissue defects. Although traditionally pioneered by plastic surgeons, microsurgery has since progressed and is increasingly being utilized by other specialities such as otolaryngology, orthopaedics and neurosurgery. With the current refinements in technique and materials, the success rates of free flaps in developed countries are as high as 97%–99%.^1^, ^2^ However, there is a growing gap between developed and developing countries, with microsurgery completely unavailable or rudimentary in many developing countries, particularly in the east, central and southern Africa (ECSA) region.^3^

Excluding South Africa, there are scarcely any reports on microsurgical free tissue transfer performed in sub-Saharan Africa. A few procedures are occasionally performed by surgeons visiting from developed countries with variable results.^4^, ^5^, ^6^, ^7^ Nevertheless, local teams most notably in Kenya and Uganda have overcome the numerous challenges and have published their experiences in a resource-limited setting not indifferent from those found in other developing countries.^3^, ^6^ Operations in the region have largely been elective reconstructive procedures most commonly for head and neck pathology, such as cancer, noma and post-burn contracture, with the most frequently utilized flaps being the radial forearm, free fibular and anterolateral thigh flaps.^3^, ^4^, ^5^, ^6^^,^^8^ Nangole et al. reported using relatively inexpensive methods in Kenya including a basic microsurgery set along with surgical loupes to perform free tissue transfers.^3^ However, such methods have attracted a mixed response of both praise and criticism from the global community.^9^

Several challenges to performing microsurgery in the region have been noted from the publications of individual units and visiting surgeons. These include poor postoperative monitoring, lack of high-quality equipment and a lack of surgical skill together resulting in relatively low free flap survival rates of 76%–89%.^3^, ^6^^,^^7^ However, there is a paucity of literature objectively assessing these challenges particularly in countries that do not often practice microsurgery. Additionally, the perceived challenges noted from individual unit experiences differ widely. For instance, Citron et al.^6^ found equivocal flap survival rates in cases performed in Uganda between local surgeons and experienced visiting surgeons from developed countries suggesting lack of surgical skill^3^ may not be the prime cause of stagnation. This highlights the crucial need to further explore the causes of suboptimal results in the region.

On the positive side, the number of plastic surgeons in the ECSA region is rapidly growing, largely due to efforts in regional cooperation of surgical training fostered by the College of Surgeons East Central and Southern Africa (COSECSA) and its partners.^10^, ^11^ Considering this, coupled with the positive economic growth seen over the last decade,^12^, ^13^ microsurgery is poised to play a greater part in reconstructive surgery in this region in the years to come.

The aim of this study was to assess the opinions of local plastic surgeons on the challenges faced practising microsurgery in the ECSA region and how to improve the service.

## Methodology

An anonymous survey (5-point Likert-style) was sent to all plastic surgeons registered with COSECSA. Additional invitations were sent to all plastic surgeons registered with respective national plastic surgery associations/societies to ensure surgeons not part of the regional college were also contacted. The countries forming the ECSA region included in this survey were; Burundi, Ethiopia, Kenya, Malawi, Mozambique, Namibia, Rwanda, South Sudan, Tanzania, Uganda, Zambia and Zimbabwe. Surgeons from Namibia were contacted individually, as the country only recently joined the regional body. A total of 57 surgeons were invited. Plastic surgeons resident and practising in the region (including academic and administrative positions) as of July 1, 2018, were included in this study. All surgeons without permanent residency in the region, such as visiting surgeons, charity missions and COSECSA overseas fellows, were excluded. Email reminders were sent after 2 weeks and 4 weeks to encourage participation.

Data were collected for country of clinical practice, years of experience, number of microsurgery procedures performed over the last 5 years, opinions on the challenges of microsurgery and suggestions for improvement. The survey was delivered through an online platform, Google Forms (https://goo.gl/forms/nKnhD1MzFNN1Gxgh1). Respondents were grouped into two groups by country of practice: countries with surgeons reporting > 10 microsurgical procedures annually were assigned to study Group A and the rest to Group B.

All survey questions were digitalised and analysed using IBM SPSS Statistics 25 (IBM Corp. Released 2017. IBM SPSS Statistics for Windows, Version 25.0. Armonk, NY: IBM Corp). Data were analysed depending on country group and surgeons' years of experience using two-tailed Mann–Whitney U and Kruskal–Wallis tests, respectively. Statistical significance was defined as *P* < 0.05.

## Results

**Figure 1:**
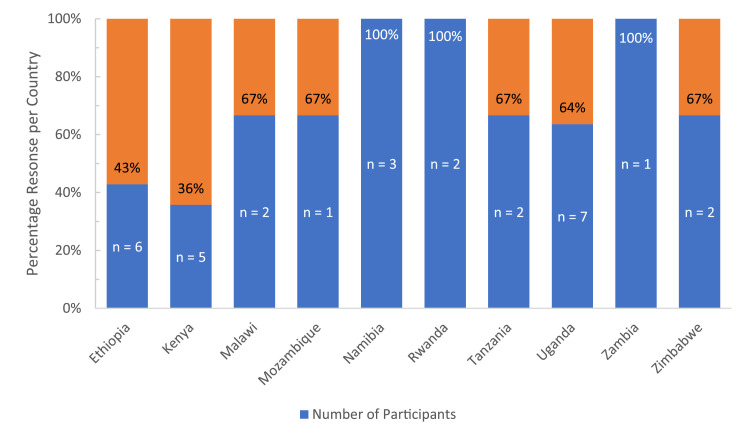
Participants by Country.

**Figure 2:**
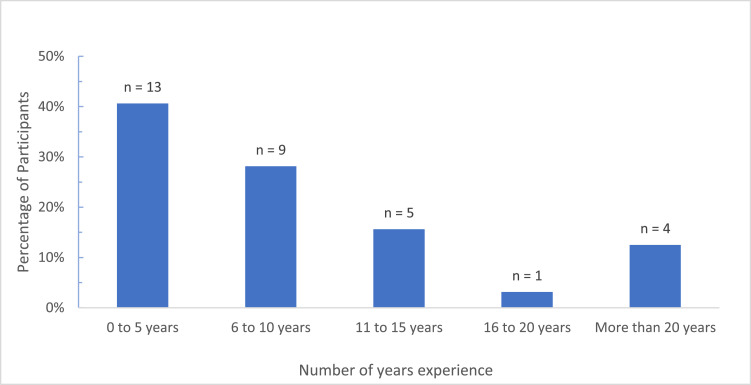
Participants by Years of Experience.

**Figure 3:**
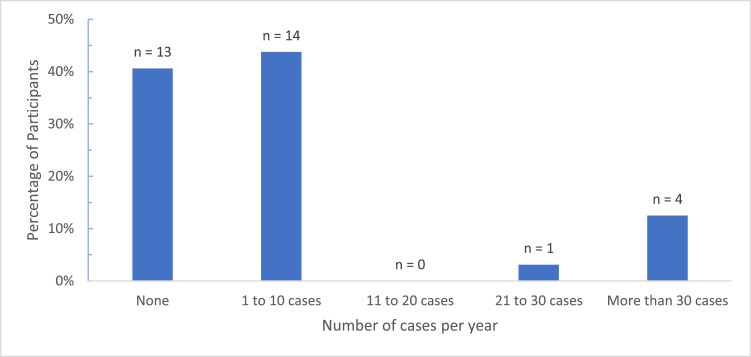
Number of Reconstructive *Microsurgical Procedures Performed (5-Year Experience)*.

The survey response rate was 56%, with 32 of the regions 57 surgeons completing the survey. All countries in the region were represented except for Burundi and South Sudan, which did not have any known plastic surgeons (Figure 1). Most respondents were young surgeons with 0–5 years of experience (Figure 2). The overwhelming majority (*n* = 31, 97%) felt that microsurgery was essential in the region, Median score (Mdn) 5, Interquartile range (IQR) 0, with a correspondingly high number (*n* = 28, 88%) interested in microsurgery (Mdn = 4.5, IQR = 1). Overall, 13 (41%) had not performed any microsurgical procedures in the past 5 years with the bulk of surgeons (*n* = 14, 44%) reporting 1–10 cases annually (Mdn = 2, IQR = 1) (Figure 3). There was no significant difference in the rate of procedures performed when compared by surgeons' years of experience (Kruskal–Wallis *H* = 2.544, *df* = 2,*P* = 0.28).

Two countries, Kenya and Uganda, were identified with surgeons performing > 10 cases annually. Accordingly, respondents from these countries were allocated to Group A (*n* = 12) and all others to Group B (*n* = 20) for subgroup analysis. Participants from the two groups were similar with regard to surgeons' years of experience (Mann–Whitney *U* = 112.5, *P* = 0.76).

Most surgeons uniformly agreed with the challenges listed, with *shortage of trained support staff* receiving the highest score (Mdn = 5, IQR = 1) with 29 (91%) in agreement (Table 1). *Lack of microsurgery materials* was an exception with highly polarized views (Mdn = 4, IQR = 3), 21 (66%) agreeing and 8 (25%) disagreeing. However, no significant difference was found when compared by surgeon experience or country group.

**Table 1:** Challenges.

Challenge	Agreement		Neutral		Disagreement		Median	IQR	*P*_experience_	*P*_country_
	n	%	n	%	n	%				
Lack of operating equipment/instruments	26	81	1	3	5	16	5	1	0.42	0.55
Lack of microsurgery materials	21	66	3	9	8	25	4	3	0.61	0.90
Limited operating theatre time	25	78	2	6	5	16	4	1	0.17	0.49
Shortage of surgical expertise	27	84	2	6	3	9	4	1	0.04	0.12
Local microsurgery training adequate	8	25	1	3	23	72	2	3	0.30	0.003
Shortage of trained support staff	29	91	1	3	2	6	5	1	0.36	0.26
Inadequate patient monitoring post-operatively	27	84	0	0	5	16	4	1	0.93	0.13
Inadequate perioperative care	25	78	4	13	3	9	5	1	0.97	0.80

**Figure 4:**
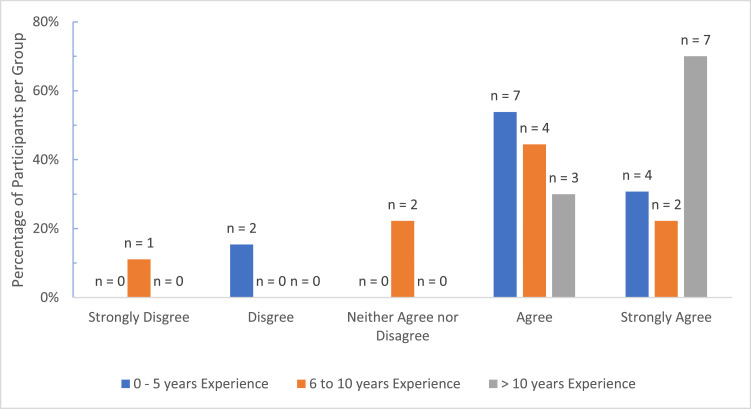
Comparison of Opinions on Shortage of Surgical Expertise by Surgeon Level of Experience.

**Figure 5:**
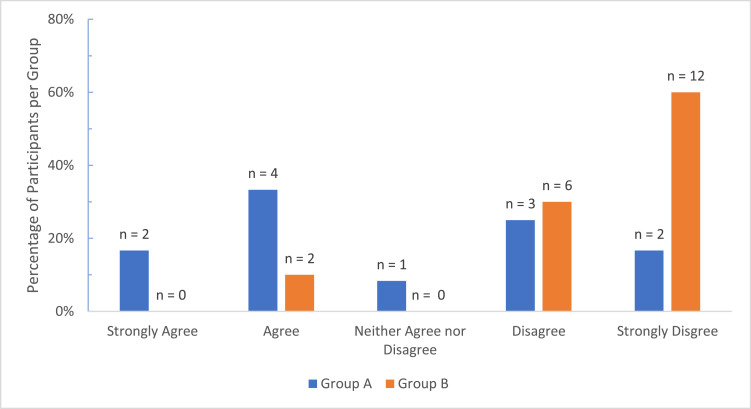
Comparison of Opinions on Adequacy of Local Microsurgery Training by Country Group.

In contrast, opinions on the *shortage of surgical expertise* differed significantly with more surgeons of >10 years of experience in agreement (*n* = 10, 100%) compared to the 6–10 years (*n* = 6, 67%) and <5 years groups (*n* = 11, 85%) (Kruskal–Wallis *H* = 6.287, *df* = 2, *P* = 0.04) (Figure 4). This difference was significantly greater between the >10 and 6–10 years groups (Mann–Whitney *U* = 19.0, *P* = 0.02). Significant disparity was also found between country group views on *Local microsurgery training* with more respondents from Group A, 6 (50%, Mdn = 3.5, IQR = 2) feeling that their local training was adequate in contrast to Group B where 18 (90%, Mdn = 1, IQR = 1) uniformly felt their local training was inadequate (Mann–Whitney *U* = 49.0, *P* = 0.003) (Figure 5).

The most common of the challenges in the unguided segment was *lack of awareness of the benefits of microsurgery* (*n* = 7, 22%), chiefly among non-plastic surgeon doctors (*n* = 5, 16%) and also among members of the public (*n* = 3, 9%) (Table 2). Various suggestions were put forward with the most frequent being *improve microsurgery training* (*n* = 19, 59%) (Table 3).

**Table 2:** Other Challenges (Unguided Responses).

Challenges	Frequency	Percentage
Inadequate training	3	9%
Lack of materials/drugs	2	6%
Inadequate operating theatre time	3	9%
High Cost/Unaffordable	4	13%
Lack of public awareness	7	22%
Lack of support from other disciplines	5	16%
Inadequate microsurgery expertise	5	16%
Others (healthcare systems and poor remuneration)	4	13%

**Table 3:** Suggestions for Improvement.

Suggestions	Frequency	Percentage
Improve microsurgery training	19	59%
More advocacy	6	19%
More funding for PRS/Microsurgery units	6	19%
Provision of materials	2	6%
Improve equipment/Infrastructure	10	31%
Set dedicated theatre time/Microsurgery units	5	16%
More microsurgery exposure	3	9%
Team building	8	25%
Others (networking and interest)	5	16%

## Discussion

Local plastic surgeons recognise that microsurgery is essential in the region and have a keen interest in improving the practice. As of 2016, the ECSA region had 46 plastic surgeons serving a population of 320 million (equivalent to the entire United States of America population), stressing the unique burden faced by plastic surgeons in this region.^10^ The predominantly young age of participants with 41% (*n* = 13) 0–5 years post residency reflects the expeditious growth of the field in the region. While the challenges related to inadequate resources such as lack of equipment and staff shortages endemic to all developing countries are well established,^3^, ^5^^,^^6^, ^9^^,^^14^ the study sought to define which ones are more compelling as well as reveal other challenges unique to this special region.

The study found that inadequate perioperative care was the main challenge faced, with all three separate questions relating to *inadequate postoperative monitoring, shortage of support staff* and *inadequate perioperative care* showing uniform agreement. This opinion was consistent across different levels of experience and country group. Good perioperative care is crucial for success in microsurgery. When absent, patient lives are endangered, and correction of complications is delayed, leading to poor results. Most surgeons (*n* = 29, 91%) attributed this to lack of trained support staff such as nurses and anaesthetists with the skill to care for microsurgery patients. This result corresponded with the published experiences of surgical units who noted that inadequate perioperative monitoring, deficient critical care and poor postoperative nursing care were key causes of poor outcomes^3^, ^6^^,^^7^.

Interestingly, opinions concerning the deficiency of microsurgical skills revealed intriguing differences. Although the majority felt local microsurgical expertise was inadequate, significantly more senior surgeons shared this view than their lesser experienced counterparts, suggesting the later could be underestimating the local skills deficit. Similarly, views on the adequacy of local microsurgery training were largely negative. However, significant contrast existed between country groups with nearly all surgeons from Group B (*n* = 18, 90%) against half from Group A (*n* = 6, 50%) feeling their local training was inadequate. This suggests that improved local training may increase microsurgery practice. In a region with a severe numerical shortage of plastic surgeons, inadequate microsurgery skill further exacerbates the problem. To address this challenge as well as poor perioperative care, it was suggested that the region improves the microsurgery training of surgeons as well as undertake a multidisciplinary team-building training approach involving not only surgeons but also other support staff.

Perhaps the least expected result was the emphasis on public lack of awareness of the benefits of microsurgery as a critical challenge. This was not previously reported in literature but was the leading response in the unguided segment of the study questionnaire. Many shared the view that unawareness primarily among the medical fraternity was at fault, extending to members of the general public and leaders. This implies that many people in the region may simply be unaware that life-changing microsurgical procedures such as extremity replantation can be performed by local plastic surgeons with support provided. In a similar manner, participants felt the necessary support from members of other surgical specialties was lacking, leading to poor coordination in patient management. Increased advocacy was proposed to correct this lack of knowledge as well as the negative perceptions of microsurgery.

Finally, the broader problem of inefficient healthcare systems in the region was noted as an important obstacle. An illustration of this is seen in the frequent delays in patient transfer across different centres, or even within the same hospital.^14^ Such obstacle, in a setting with limited operating theatre time, makes microsurgery impractical. Although the establishment of national or regional microsurgery units that would also serve as training hubs was proposed, the resolution of this problem goes far beyond the influence of individual plastic surgery units. It requires institutional, national and regional policy change achievable possibly through global surgery advocacy.

While this study focused on ECSA, it provides useful insight into the challenges faced in reconstructive microsurgery in the wide sub-Saharan region. In many aspects, the countries studied share the same disease burden as well as health sector financial and human resource restraints as the many other low- and middle-income countries across the continent.

The study was limited by a poor response particularly from Kenya, which alone accounts for a quarter of the regions’ plastic surgeons. This could be attributed to the general low participation of Kenyan plastic surgeons in regional affairs, with only 3 of the 14 plastic surgeons registered with the regional college COSECSA. Additionally, the use of a structured questionnaire may have restricted respondents from fully expressing their opinions. To mitigate this, we included two open-ended questions allowing participants to freely share their views. Finally, the study canvassed the opinions of individual surgeons without including the hospitals where each are based. This is important because in most parts of Africa, the medical services differ widely between urban and rural centres, as well as across public, private and mission facilities. To address this, further study on a situational analysis for each country may be required for a more comprehensive evaluation. The regional surgical college could spearhead this evaluation. However, local plastic surgeons need to lead in advocacy and share their experiences in reconstructive microsurgery in this unique part of the world. This in turn will foster advancement of innovations and techniques that work best for the population in this region, thereby improving outcomes.

In conclusion, as we look to a future of increased microsurgery practice in sub-Saharan Africa, the key challenges in perioperative care, surgical expertise and public lack of awareness need to be addressed alongside resource allocation to improve outcomes. This can be achieved through enhanced training, multidisciplinary team building and advocacy both locally and on a global scale.

## Conflict of interest

The authors declare that there is no conflict of interest.
